# Decoding the Risks of Fecal Incontinence: A Comprehensive Analysis of Closed Internal Sphincterotomy in Chronic Anal Fissure

**DOI:** 10.7759/cureus.74216

**Published:** 2024-11-22

**Authors:** Babur Farid, Haseena Rehman, Mukhtiar Ali, Muska Miraj, Mahnoor Amjad, Basit Ali, Zarak Khan, Muhammad Abu Bakkar

**Affiliations:** 1 Medicine and Surgery, Musgrove Park Hospital, Taunton, GBR; 2 General Surgery, Lady Reading Hospital Medical Teaching Institution (MTI), Peshawar, PAK; 3 Urology, Institute of Kidney Diseases, Hayatabad Medical Complex, Peshawar, PAK; 4 General Surgery, Rehman Medical Institute, Peshawar, PAK

**Keywords:** anal fissure, chronic anal fissure, closed internal sphincterotomy, closed lateral internal sphincterotomy, faecal incontinence

## Abstract

Introduction

An anal fissure is marked by a longitudinal tear in the mucosal lining of the lower anal canal, causing painful defecation and mild anal bleeding. The classical triad includes an anal ulcer, a sentinel tag, and a hypertrophic papilla.

This study investigates the frequency of fecal incontinence in patients with anal fissure undergoing closed internal sphincterotomy, offering recent insights for treatment recommendations.

Objective

To determine the prevalence of fecal incontinence in individuals with chronic anal fissure undergoing closed internal sphincterotomy.

Methodology

The study design was a descriptive case series, conducted over a 6-month period (August 21, 2018 to February 21, 2019). It was carried out at the General Surgery Department, Lady Reading Hospital MTI, Peshawar, Pakistan. The participants included a total of 139 patients diagnosed with chronic anal fissures.

Data collection

To gather comprehensive information, a detailed approach was adopted. This included history taking, general physical examinations, and digital rectal examinations for all patients. All patients diagnosed with chronic anal fissure were prepared for lateral internal sphincterotomy (LIS).

Variables

The variable of interest was the occurrence of fecal incontinence, assessed during follow-up visits at the end of the 2nd and 6th week post-surgery.

Ethical consideration

The study received approval from the hospital's ethical committee and the College of Physicians and Surgeons of Pakistan (CPSP) research committee.

Results

In our study, the mean age was 30 years (SD ± 12.16). Forty-five percent of patients were male, and 55% were female. Fecal incontinence was observed in 10% of patients.

Conclusion

Our study reveals a fecal incontinence frequency of 10% in patients undergoing closed LIS for chronic anal fissure.

## Introduction

An anal fissure is marked by a longitudinal tear in the mucosal lining of the lower anal canal, causing painful defecation and mild anal bleeding. The classical triad includes an anal ulcer, sentinel tag, and hypertrophic papilla. The exact cause is not known; however, high sphincter pressure and secondary local ischemia are suggested as causative factors [1]. It is triggered by stretching trauma, such as the passage of sizable or firm bowel movements and episodes of forceful diarrhea [2,3]. A superficial fissure can naturally heal on its own. However, in the case of a deep tear, compromised blood supply to the covering mucosa caused by sphincter spasms can lead to a persistent non-healing fissure, which has the potential to become infected with fecal bacteria [4]. There is no strict agreement on how long a fissure needs to last to be considered chronic, but most sources suggest a range of about four to twelve weeks [5]. The exact prevalence of fissures is unknown, but research indicates that up to 20% of individuals may experience a fissure at some point in their lives [6].

The treatment for anal fissure focuses on alleviating internal anal sphincter spasms through either chemical relaxation or surgical intervention. Medical interventions include applying glyceryl trinitrate (GTN), using nifedipine and diltiazem ointments, and administering botulinum toxin injections [7]. GTN induces relaxation in both the sphincter and blood vessels, thereby diminishing sphincter spasms. This action enhances blood supply to the mucosa covering the fissure, facilitating the healing process [8].

Lateral internal sphincterotomy (LIS) is considered the primary approach for the definitive treatment of anal fissures, demonstrating a fissure healing rate of 96.5%. However, it is associated with the potential risk of incontinence [7,9]. During a LIS, a cut is made in the lower third of the sphincter through either an incision or a stab around the anal verge. This procedure is associated with a lower recurrence rate, but common complications include pain, bleeding, and sepsis. Additionally, there may be transient urinary retention, incontinence, and the possibility of recurrence as side effects [10]. Patients undergoing this procedure are cautioned about the potential risk of incontinence, although there is limited information on long-term outcomes. In a study, the comprehensive complication rate for LIS in treating anal fissure was reported to be 13.2% [11]. In a separate study, persistent pain was noted in 10% of patients after undergoing LIS. Additionally, bleeding occurred in 8% of cases, wound infection in 10%, and fissure recurrence in 6% of patients. Flatus incontinence and mild fecal soiling were reported in 6% and 10% of patients, respectively [12]. Post-operative fecal incontinence in patients who underwent closed lateral internal anal sphincterotomy (CLIAS) was 4.3%, significantly lower than the 21.3% observed in those undergoing open internal anal sphincterotomy (OIAS), with a p-value of 0.027. The use of CLIAS with the von-Greaves knife proved effective in reducing fecal incontinence on the 5th postoperative day compared to the standard OIAS procedure [13]. CLIAS with the von-Greaves knife demonstrates effectiveness in reducing fecal incontinence on the 5th postoperative day compared to OIAS, specifically Park's procedure. Consequently, considering this positive outcome, the CLIAS technique may be regularly employed in the future for the treatment of chronic anal fissure to mitigate the risk of fecal incontinence [14].

## Materials and methods

This descriptive case series study was conducted at the Department of General Surgery, Lady Reading Hospital, Peshawar, over a period of 6 months from September 8, 2018 to February 9, 2019. The sample size was calculated to be 139 using the WHO software for power analysis, based on a 10% proportion of fecal incontinence after LIS for anal fissure, with a 95% confidence level and 5% absolute precision. A consecutive (non-probability) sampling technique was employed for patient selection.

Inclusion criteria

The inclusion criteria for this research were carefully designed to ensure a focused and representative sample of patients with chronic anal fissures. It included patients who had failed conservative medical interventions such as GTN, nifedipine and diltiazem ointments, and botulinum toxin injections. The study included both male and female patients within the age range of 18 to 60 years diagnosed with uncomplicated chronic anal fissures. Specifically, only patients with uncomplicated chronic anal fissures were eligible for inclusion in the study. The age range of 18 to 60 years was chosen to focus on adult patients while excluding older individuals who might have additional age-related complications. The criterion of 'uncomplicated chronic anal fissure' is crucial, as it helps maintain a homogeneous study population. Uncomplicated cases typically refer to anal fissures not associated with other anorectal conditions or systemic diseases that could confound treatment outcomes.

Exclusion criteria

To ensure a focused and representative sample, several exclusion criteria were applied. Patients with known cases of intestinal tuberculosis, anorectal carcinoma, ulcerative colitis, or Crohn's disease were excluded based on medical records and medication history. Conditions such as Nicorandil use, complicated vaginal delivery, neuromuscular disorders (multiple sclerosis, spinal trauma, motor neuron disease, etc.), previous ano-rectal surgery affecting the functioning of the sphincter complex, digital rectal examination findings, or colonoscopy results were also criteria for exclusion. Additionally, individuals presenting with associated perianal abscesses, as determined by clinical examination, were not included in the study. Patients undergoing anticoagulant therapy were excluded due to the increased risk of bleeding. The study also omitted known diabetics with fasting blood sugar levels exceeding 126 mg/dl and obese individuals with a BMI greater than 30 kg/m².

Including the above-mentioned conditions would have introduced bias in the study results.

Data collection

This study was conducted after obtaining approval from both the hospital's ethical committee and the CPSP research committee. Eligible patients were recruited through the Outpatient and Surgical Departments following a comprehensive explanation of the study's objectives and potential benefits. Each participant provided written informed consent prior to enrollment.

Data collection was thorough and multifaceted. We obtained detailed medical histories, performed comprehensive physical examinations including digital rectal examinations, and conducted routine investigations for all patients. The diagnosis of anal fissure was established based on the clinical observation of a vertical breach in the lower anal mucosa.

All participants underwent LIS within 24 hours of approval. The procedures were performed by four consultants with a minimum of five years of surgical experience and CPSP fellowship. The closed LIS technique involved carefully introducing a surgical blade between the internal and external anal sphincters, palpating the intersphincteric groove at the 3 o'clock position with the patient in the lithotomy position. The internal anal sphincter was then incised up to the dentate line, leaving the external wound open.

Post-operative care included discharge on the second day with prescriptions for oral antibiotics and analgesics for a two-week course. Patients were also advised to follow a regimen of sitz baths and use stool softeners for six weeks. Follow-up visits were scheduled at 2 and 6 weeks post-surgery, primarily to assess for fecal incontinence. Patients who did not complete the follow-up protocol were excluded from the final analysis.

To ensure data accuracy and completeness, all relevant patient information, including demographic data, was meticulously recorded using a pre-designed proforma. We maintained strict adherence to our predefined inclusion criteria throughout the study to minimize potential confounders and biases that could affect the validity of our results.

Data analysis

Data analysis was conducted using IBM SPSS Statistics version 22. Descriptive statistics were employed to summarize the study variables. For quantitative variables, such as age, measures of central tendency and dispersion were calculated, specifically the mean and standard deviation. Qualitative variables, including gender and the presence of fecal incontinence, were described using frequencies and percentages. To explore potential effect modifiers, fecal incontinence was stratified by age and sex. Post-stratification analysis utilized the Chi-square test to assess the significance of associations between variables. The threshold for statistical significance was established at p-value ≤ 0.05. To enhance the interpretability of the results, various visual representations were generated. These included tables summarizing descriptive statistics and frequency distributions, as well as appropriate charts and graphs to illustrate key findings. The visual aids were carefully designed to provide a clear and concise overview of the data, facilitating a more intuitive understanding of the study outcomes. This comprehensive analytical approach allowed for a thorough examination of the data, enabling the identification of patterns and relationships that might not be immediately apparent from raw data alone. The combination of statistical tests and visual representations provided a robust foundation for drawing meaningful conclusions from the study results.

## Results

Our study encompassed a total of 139 patients who underwent closed lateral internal sphincterotomy for chronic anal fissure. The age distribution of the participants revealed a diverse range, with the majority falling within the middle-age brackets. Specifically, 29 patients (21%) were aged 18-30 years, 42 patients (30%) were 31-40 years, 46 patients (33%) were 41-50 years, and 22 patients (16%) were 51-60 years. The mean age of the study population was 30 years, with a standard deviation of ±12.16 years, indicating a relatively young to middle-aged cohort (Table 1).

**Table 1:** Age distribution. Mean age was 30 years with SD ± 2.16

Age	Female frequency	Female percentage	Male frequency	Male percentage
18-30 years	18	23.68%	11	17.46%
31-40 years	22	28.94%	20	31.74%
41-50 years	23	30.26%	24	38.95%
51-60 years	13	17.10%	8	12.69%
Total	76	54.68	63	45.32

Regarding the primary outcome of our study, fecal incontinence was observed in 14 patients (10%) following the procedure, while 125 patients (90%) did not experience this complication (Table 2). This incidence rate aligns with previous studies in the literature, suggesting that closed lateral internal sphincterotomy is generally well-tolerated, with a minority of patients experiencing post-operative fecal incontinence.

**Table 2:** Fecal incontinence (n=139).

Fecal incontinence	Frequency	Percentage
Yes	14	10%
No	125	90%
Total	139	100%

To elucidate potential risk factors for fecal incontinence, we stratified our results by age and gender. The age-stratified analysis (Table 3) showed that fecal incontinence occurred across all age groups, with a slight trend towards a higher incidence in the middle age brackets (31-50 years). However, the chi-square test yielded a p-value of 0.9949, indicating no statistically significant association between age and the occurrence of fecal incontinence in our sample.

**Table 3:** Stratification of fecal incontinence with age (n=139). Chi square test was applied in which p-value was 0.9949.

Fecal incontinence	18-30 years	31-45 years	41-50 years	51-60 years	Total
Yes	3	4	5	2	14
No	26	38	41	20	125
Total	29	42	46	22	139

Gender stratification (Table 4) revealed that among the 14 patients who experienced fecal incontinence, 6 were male and 8 were female. This distribution closely mirrored the overall gender distribution of our study population (63 males and 76 females). The chi-square test resulted in a P-value of 0.8449, suggesting no significant gender-based difference in the risk of post-operative fecal incontinence.

**Table 4:** Stratification of fecal incontinence with gender (n=139). Chi-square test was applied in which p-value was 0.8449.

Fecal incontinence	Male	Female	Total
Yes	6	8	14
No	57	68	125
Total	63	76	139

To enhance the robustness of our findings, we employed the Wexner score, a validated tool for assessing the severity of fecal incontinence, to evaluate the patients who experienced this complication post-operatively. Among the 14 patients who developed fecal incontinence following the procedure, 11 were classified as having moderate incontinence, while 3 patients presented with severe incontinence according to the Wexner scoring system.

It is worth noting that our study population had a slightly higher proportion of female patients (76 out of 139, or 54.7%) compared to males (63 out of 139, or 45.3%). This gender distribution is consistent with some previous studies that have reported a higher prevalence of anal fissures among women.

These results provide valuable insights into the outcomes of closed lateral internal sphincterotomy for chronic anal fissure in our patient population. The overall low incidence of fecal incontinence, coupled with the lack of significant age or gender-based risk factors, supports the safety and efficacy of this procedure. However, the 10% incidence of fecal incontinence underscores the importance of careful patient selection and thorough pre-operative counseling. Furthermore, the Wexner assessment provides a more nuanced understanding of the impact of the surgical intervention on patient outcomes. These findings underscore the importance of comprehensive post-operative evaluation and highlight the need for tailored follow-up care for patients experiencing varying degrees of fecal incontinence.

## Discussion

Anal fissure, though common, often goes undiagnosed and can be mistaken for hemorrhoids. The hallmark symptom is pain during and after defecation, which can be severe, leading some patients to avoid bowel movements. The pain is accompanied by rectal bleeding in many cases. While acute fissures may resolve with dietary adjustments, chronic cases often require intervention.

The evolution of anal fissure treatment has shifted from surgical to medical approaches, both aimed at alleviating internal anal sphincter spasm [1]. The anal canal's poor perfusion, particularly in the posterior midline, exacerbates relative ischemia, especially when compounded by sphincter spasm [15]. Manual dilation, historically a primary treatment for chronic fissure, carried risks of uncontrolled tearing and incontinence [16].

LIS has demonstrated superiority over anal dilation, albeit with documented risks of incontinence [17].

Efforts to mitigate sphincterotomy risks include tailored procedures and conservative division cephalad to the fissure's length. Our study, involving 139 patients, aligns with previous research, showing 10% experiencing fecal incontinence. Comparable figures were found in other studies, reinforcing the overall complication rate of LIS [11].

Postoperative pain reduction in our study, echoing findings in another study [18], indicates significant, though not complete, pain relief. This corresponds with similar outcomes observed in studies conducted by Muhammad Saleem Arshad, Aamer Zaman Khan, et al. [19].

Fissure healing in our study at the end of the 6th week was 94.3%, slightly lower than in other studies [20], likely due to shorter follow-up periods and rural patients' habits affecting wound healing. Our study introduces novel variables: bleeding cessation (97.1%) and overall satisfaction (95%) at 6 weeks postoperative. Local wound infection occurred in 5.7%, impacting symptom persistence.

LIS emerges as an effective treatment for anal fissure, offering quick relief and high patient satisfaction. Mitigating risks involves postoperative care, such as stool softeners and fiber supplementation. While fecal soiling and incontinence risks exist, meticulous surgical techniques and postoperative measures can address these concerns effectively. The study underscores the importance of tailoring interventions to patient demographics and local practices, providing valuable insights for future treatment strategies.

Limitation

This study, while providing valuable insights into fecal incontinence following LIS for chronic anal fissures, acknowledges several limitations that warrant consideration. Firstly, the sample size of 139 patients, observed over a six-month period, may limit the generalizability of our findings. A larger cohort studied over an extended timeframe could potentially yield more robust and widely applicable results. Additionally, our research would have benefited from a more comprehensive assessment of the quality of life impact associated with post-operative fecal incontinence. Incorporating validated quality of life measures could have provided a more nuanced understanding of the procedure's overall effects on patient well-being. Lastly, we recognize the potential for reporting bias in our study. The sensitive nature of fecal incontinence may have led to underreporting by some patients, possibly due to embarrassment or social stigma. This could have influenced the accuracy of our reported incidence rates. Future research in this area should consider addressing these limitations to further enhance our understanding of the outcomes following LIS for chronic anal fissures.

## Conclusions

In conclusion, our study highlights a fecal incontinence frequency of 10% among patients undergoing closed internal sphincterotomy for chronic anal fissure. This finding contributes to the understanding of the outcomes associated with this surgical intervention, emphasizing the importance of careful consideration and patient counseling regarding potential incontinence risks. Further research and long-term follow-ups are warranted to refine our understanding of the outcomes and guide clinical decision-making in the management of chronic anal fissure.

## Figures and Tables

**Figure 1:**
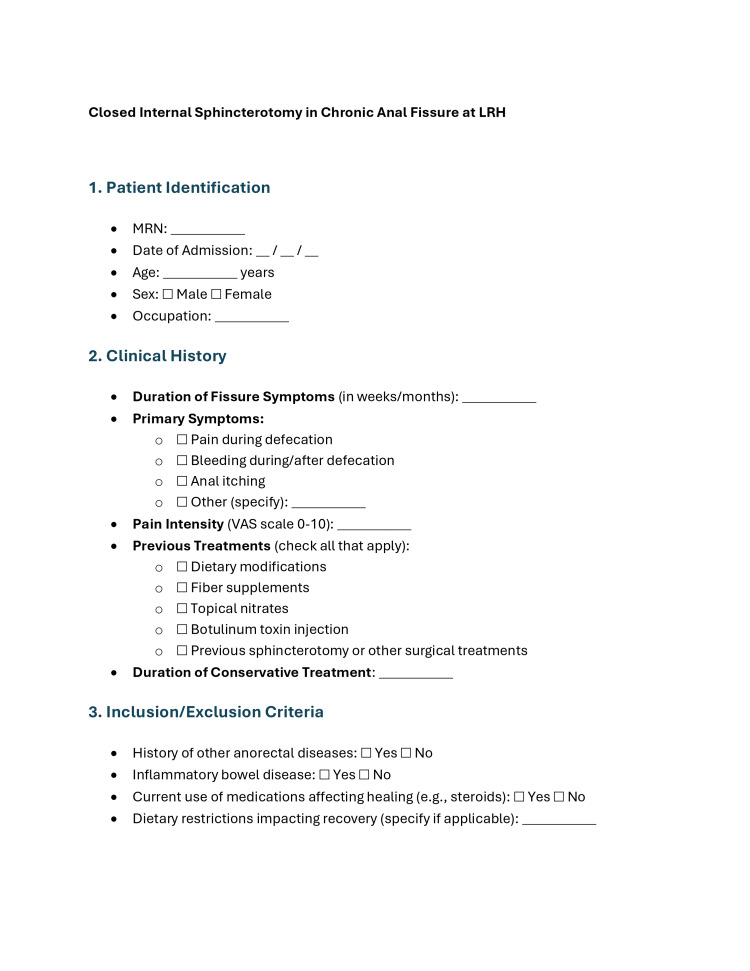
Proforma page 1. Proforma used for data collection.

**Figure 2:**
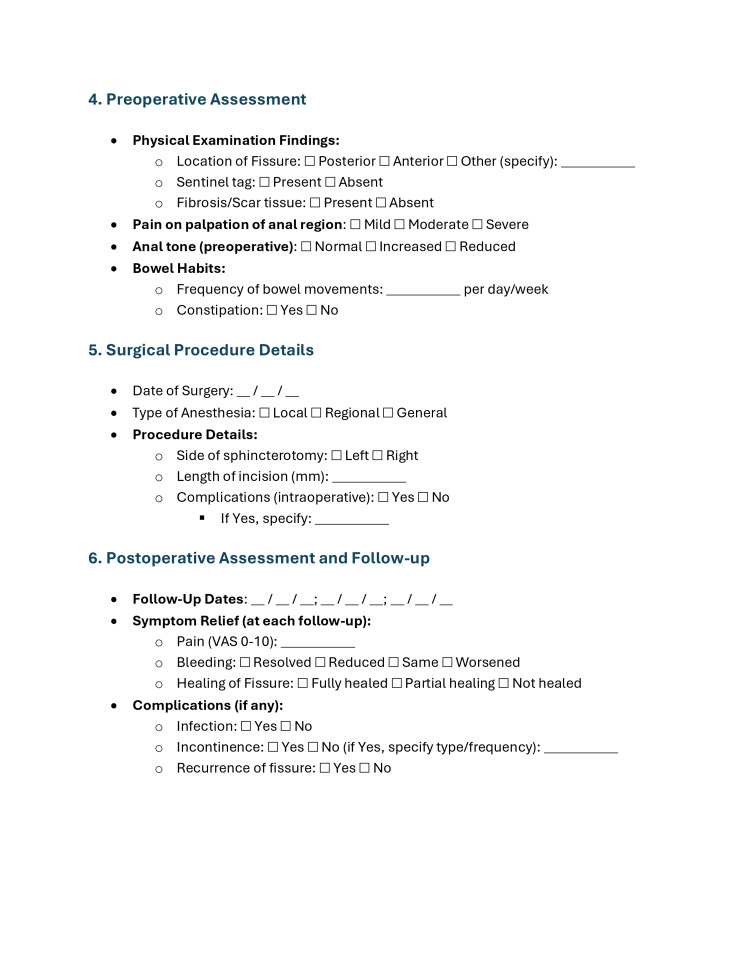
Proforma page 2. Data collection proforma page 2

**Figure 3:**
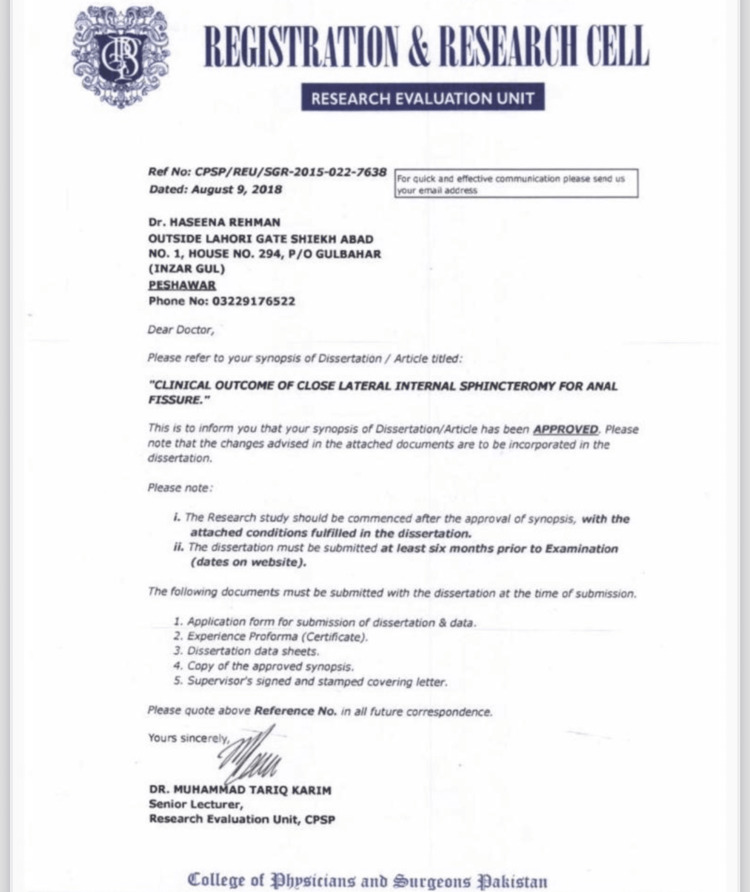
IRB approval. The approval from College of Physicians and Surgeons of Pakistan (CPSP)'s Registration and Research Cell for commencing with this research project.

## Appendices

Appendix 1
